# Prognostic value of the left ventricular ejection fraction reserve acquired by gated myocardial perfusion SPECT in patients with CAD and reduced stress LVEF

**DOI:** 10.3389/fcvm.2024.1480501

**Published:** 2024-10-10

**Authors:** Shuang Zhang, Jingjing Meng, Yihan Zhou, Lijun Lv, Xiaoli Zhang

**Affiliations:** ^1^ Department of Nuclear Medicine, Beijing Anzhen Hospital, Capital Medical University, Beijing, China; ^2^ Department of Ultrasonography, Beijing Anzhen Hospital, Capital Medical University, Beijing, China; ^3^ Medical Records Statistics Room, Beijing Anzhen Hospital, Capital Medical University, Beijing, China

**Keywords:** ejection fraction reserve, myocardial perfusion imaging, gated SPECT, prognosis, coronary artery disease

## Abstract

**Purpose:**

Left ventricular ejection fraction (LVEF) strongly predicts cardiac events. However, conflicting findings exist regarding the prognostic value of the LVEF reserve (ΔLVEF) when measured by gated single-photon emission computed tomography myocardial perfusion imaging (SPECT G-MPI). In particular, data related to the prognostic value of ΔLVEF when measured by SPECT in patients with reduced LVEF are scarce. In this study, we aimed to evaluate the prognostic value of ΔLVEF when acquired by SPECT G-MPI in patients with coronary artery disease (CAD) and a LVEF_Stress_ < 60%.

**Methods:**

We retrospectively recruited 260 consecutive patients diagnosed with CAD by coronary angiography (CAG) and a LVEF_Stress_ < 60%, as determined by SPECT G-MPI. These patients were followed up for 33.4 ± 7.6 months. The patients were divided into two groups (ΔLVEF > 0% and ΔLVEF ≤ 0%), and survival analyses were conducted. The primary endpoints were major adverse cardiac events (MACEs), a composite of all-cause death, nonfatal myocardial infarction, unplanned coronary revascularization, and hospitalization for unstable angina.

**Results:**

We observed 69 MACEs (26.5%). The cumulative incidence of MACEs in patients with ΔLVEF ≤ 0% was significantly higher than in patients with ΔLVEF > 0% (*P* = 0.042). Multivariate Cox regression further revealed that a ΔLVEF ≤ 0% represented an independent predictor of MACEs (adjusted hazard ratio [HR]: 1.276; 95% confidence interval [CI]: (1.006, 1.618), *P* = 0.045). Adding a ΔLVEF ≤ 0% to traditional myocardial perfusion and function variables evaluated by MPI significantly improved the ability to predict MACEs (*P* = 0.044).

**Conclusions:**

Determining ΔLVEF by SPECT G-MPI was associated with MACEs and improved risk stratification compared to prediction models based on traditional perfusion and functional parameters in CAD patients with left ventricular dysfunction, particularly those with no or mild myocardial ischemia.

## 1 Introduction

Coronary artery disease (CAD) is associated with high morbidity and mortality rates worldwide (1). Prognostic assessment is critical when deciding to treat patients with CAD and formulating prevention strategies. The main method used for the stratification of risk among patients with CAD is the evaluation of stress-induced myocardial ischemia, often by single-photon emission computed tomography (SPECT) myocardial perfusion imaging (MPI) (2). Essentially, gated MPI (G-MPI) enables the simultaneous assessment of the distribution of myocardial perfusion and cardiac function. Risk stratification can be enhanced by applying multiple parameters acquired by G-MPI, including myocardial perfusion data and functional information. A range of key factors, such as enlarged ventricular volume, the presence of transient ischemic dilatation (TID), and, in particular, reduced left ventricular ejection fraction (LVEF), have been identified as independent risk factors for adverse outcomes in patients with CAD (3).

LVEF is the preferred variable for evaluating LV systolic function (4). Furthermore, a reduction in LVEF reserve (ΔLVEF), defined as LVEF_Stress_ minus LVEF_Rest_ (5), has been associated with ischemic contractile dysfunction (6, 7). Previous studies utilizing ^82^Rb positron emission tomography (PET) myocardial perfusion imaging demonstrated that ΔLVEF represented an independent predictor of major adverse cardiac events (MACEs) (5, 8). Nevertheless, the prognostic value of ΔLVEF, as measured by SPECT G-MPI (9–11) has yet to be fully evaluated. Besides, most studies did not specifically focus on patients with cardiac dysfunction (10–12). Furthermore, the incremental prognostic value of an abnormal ΔLVEF in patients with reduced LVEF_Stress_ has yet to be investigated. In addition, research has shown that the extent and severity of myocardial ischemia can both influence the prognosis and a large area of ischemia (>10%/LV) is considered to be a key indicator of revascularization for patients with CAD (13). There is a significant paucity of data relating to the prognostic value of *Δ*LVEF in patients with varying degrees of myocardial ischemia, especially in patients with no or mild myocardial ischemia.

Therefore, this study aimed to evaluate the prognostic value of ΔLVEF, as determined by SPECT G-MPI, in patients diagnosed with CAD and in a high-risk group of patients with left ventricular dysfunction (LVEF_Stress_ < 60%). In addition, we analysed the prognostic value of *Δ*LVEF in patients with different degrees of myocardial ischemia.

## 2 Methods

### 2.1 Study population

Between October 2016 and December 2019, we retrospectively screened the medical records of all consecutive patients attending Anzhen Hospital for suspected CAD and who had undergone stress-rest SPECT G-MPI and a subsequent invasive coronary angiogram (CAG).

The British Society of Echocardiography recently defined the normal reference interval for LVEF as ≥ 55% (14). Reference values of LVEF are unlikely to be universally applicable across different imaging modalities and may vary among ethnic groups. According to our recent study (15), we treated a LVEF_Stress_ < 60% on SPECT G-MPI as indicative of impaired left ventricle systolic function.

Patients were included if they had: (1) a LVEF_Stress_ < 60% on SPECT G-MPI, (2) underwent invasive CAG within three months of SPECT G-MPI, and (3) had significant stenosis of the left main coronary artery and/or stenosis of at least one major coronary artery. The ethics committee of Anzhen Hospital approved the study protocol.

### 2.2 Coronary angiography

CAG was performed using either the femoral or radial approach using the standard Judkins method. Two experienced interventional cardiologists blinded to the study's objective and design performed an analysis of the Arteriography. Significant stenosis was defined as luminal narrowing ≥50% in the left main coronary artery and/or ≥70% in the major epicardial coronary arteries. Stenosis in the left main stem was defined as a two-vessel disease. Decisions relating to revascularization, as well as the choice of revascularization method, were made at the discretion of the cardiologist.

### 2.3 SPECT G-MPI

All patients underwent SPECT G-MPI following the two-day stress/rest protocol described in our previous study (16). Stress was induced by physical exertion on an ergometer bicycle or by pharmacological intervention with adenosine. In this protocol, 99mTc-sestamibi (radiochemical purity > 95%, injected dose of 740–925 MBq) was administered intravenously at peak stress. Perfusion images were captured over 8 min using a dual-headed Siemens Camera (Siemens Symbia Intevo 16 Systems) with a multifocal (SMART ZOOM) collimator. Images were reconstructed using flash 3D mode and displayed as horizontal short-axis and vertical long-axis slices.

A 17-segment model was applied by two experienced physicians who were unaware of the clinical data (17). Next, the total perfusion defect (TPD), which represents the total extent of reversible (ischemia) and fixed (scar) defects, was quantified and expressed as a percentage of the involved left ventricle.

Quantitative ECG-gated SPECT was analysed by QGS software (Cedars Sinai Medical Center, Los Angeles, CA, USA). The LVEF, end-systolic volume (ESV), and end-diastolic volume (EDV) were calculated post-stress and at rest. Subsequently, we calculated ΔLVESV (ΔLVESV = LVESV_Stress_ - LVESV_Rest_), ΔLVEDV (ΔLVEDV = LVEDV_Stress_ - LVEDV_Rest_), and ΔLVEF (ΔLVEF = LVEF_Stress_ - LVEF_Rest_). As reported previously, an abnormal LVEF reserve was defined as ΔLVEF ≤ 0% (11, 18–20). TID was described as a stress/rest left ventricle volume ratio ≥ 1.2 (21), including EDV and ESV (TIDEDV and TIDESV).

### 2.4 Follow-up

Follow-up was performed by consulting the electronic medical record system in the hospital and by contacting patients or their relatives by telephone. The primary outcome was the occurrence of MACEs, including all-cause death, nonfatal myocardial infarction, unplanned coronary revascularization, and hospitalization for unstable angina (22). Patients were censored after the first event or at the end of the follow-up period. During the follow-up period, unplanned coronary revascularization is defined as any unexpected coronary revascularization, including percutaneous coronary intervention (PCI) and coronary artery bypass graft surgery (CABG). We identified a diagnosis of unstable angina according to the ESC guidelines (13), and an expert was consulted when uncertain of a diagnosis.

### 2.5 Statistical analysis

Normally distributed continuous variables are presented as mean ± standard division, while non-normally distributed continuous variables are presented as median and interquartile range (Q1 to Q3). Categorical variables are presented as numbers (%). For all continuous variables, means were evaluated by the unpaired *t-test* or the Mann-Whitney *U*-test. Categorical variables were compared between groups using the chi-squared test or Fisher's exact test, as appropriate.

The cumulative incidence of MACEs was estimated using the Kaplan-Meier method and compared using the log-rank test. Landmark analyses were performed using a landmark point of 2 year and beyond 2 years. Independent prognostic factors associated with MACEs were determined by univariate and multivariate Cox regression, performed stepwise backward. The ΔLVEF ≤ 0% was incorporated as a time-varying covariate in Cox models. All variables were first assessed by univariate Cox proportional hazards regression analysis. Only variables with a statistically significant association with the cumulative incidence of MACEs (*P* < 0.05) were included in the multivariate model. Results are presented as hazard ratios (HRs) and 95% confidence intervals (95% CIs). In addition, we evaluated the incremental prognostic value of predicting MACEs by MPI results and LVEF reserve in comparison baseline, including age, sex and body mass index (BMI), based on calculated global *χ*^2^ values. *P* < 0.05 was defined as statically significant. All data were analysed using SPSS version 26 for Windows (IBM SPSS Statistics 26; NY, USA).

## 3 Results

### 3.1 Baseline clinical characteristics

**Figure 1:**
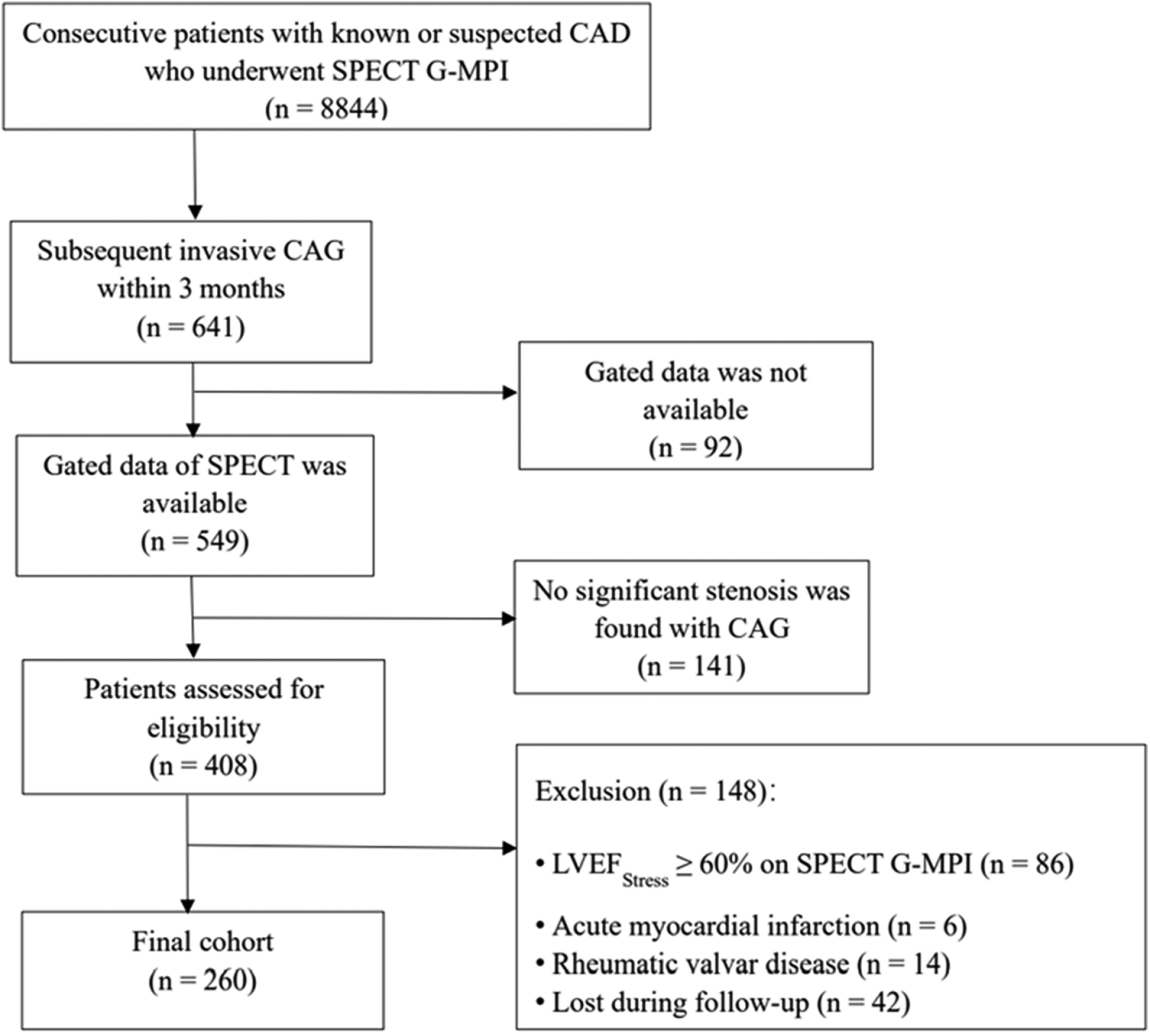
Flow diagram showing initial selection of cohort and excluded patients. CAD, coronary artery disease; SPECT G-MPI, gated single-photon emission computed tomography myocardial perfusion imaging; CAG, coronary angiography; LVEF, left ventricular ejection fraction.

A total of 8,844 consecutive patients with known or suspected CAD who underwent SPECT G-MPI were preliminarily enrolled. Among these patients, only 641 underwent invasive coronary angiography within three months. Moreover, the gated data of 92 patients was unavailable, and no significant stenosis was found in 141 patients. Additionally, from the 408 patients who were eligible for analysis, we excluded 148 patients for one of the following reasons: (1) LVEF_Stress_ ≥ 60% on SPECT G-MPI (*n* = 86), (2) acute myocardial infarction (MI) (< 8 weeks, *n* = 6), and (3) rheumatic valvar disease (*n* = 14). In addition, 42 patients (10%) were lost during follow-up. Thus, 260 consecutive patients were finally enrolled in the final analysis (Figure 1).

Of the 260 patients (age 60.4 ± 10.0 years, 206 male), 76 had an ΔLVEF > 0% and 184 had an ΔLVEF ≤ 0%. The baseline characteristics of the two groups are reported in Table 1. There was no significant difference (*P* > 0.05) between the two groups in terms of baseline characteristics, including age, gender, BMI, hypertension, diabetes, hyperlipidaemia, current smoker status, and previous revascularization. Compared with patients with an ΔLVEF > 0%, a history of prior myocardial infarction was more common in patients with an ΔLVEF ≤ 0% (*P* = 0.015).

**Table 1:** Baseline clinical characteristics of the patients.

	All	ΔLVEF > 0%	ΔLVEF ≤ 0%	*P*-value
	*n* = 260	*n* = 76	*n* = 184	
Age (years)	60.4 ± 10.0	61.1 ± 9.9	60.1 ± 10.1	0.466
Male/female	206/54	62/14	144/40	0.549
BMI (kg/m2)	26.1 ± 3.4	26.3 ± 3.1	26.0 ± 3.5	0.486
Risk factors, *n* (%)				
Hypertension	174(0.67)	52(0.68)	122(0.66)	0.741
Diabetes	100(0.39)	26(0.34)	74(0.40)	0.365
Hyperlipidaemia	151(0.58)	42(0.55)	109(0.59)	0.555
Current smoker	85(0.33)	25(0.33)	60(0.33)	0.964
Previous infarction	60(0.23)	10(0.13)	50(0.27)	**0.015**
Previous revascularization	73(0.28)	16(0.21)	57(0.31)	0.105
Stressor				0.229
Exercise	68(0.26)	16(0.21)	52(0.28)	
Regadenoson	192(0.74)	60(0.79)	132(0.72)	
SPECT G-MPI results				
Total perfusion defect (%)	18(10, 30)	22(12, 35)	18(9, 28)	0.070
Scar extent (%)	0(0, 12)	0(0, 15)	0(0, 12)	0.577
Ischemia extent (%)	12(0, 18)	12(0, 24)	12(1, 18)	0.445
Ischemia extent > 10%, *n* (%)	141(0.54)	41(0.54)	100(0.54)	0.953
LVEDV_Stress_ (ml)	93(77, 109)	98(83, 111)	91(75, 106)	0.050
LVESV_Stress_ (ml)	45(37, 56)	45(39, 54)	44(37, 57)	0.676
LVEF_Stress_ (%)	51(45, 55)	54(48, 57)	51(44, 54)	**0.001**
LVEDV_Rest_ (ml)	86(73, 105)	92(74, 107)	85(72, 103)	0.161
LVESV_Rest_ (ml)	42(33, 53)	46(38, 56)	39(32, 50)	**0.001**
LVEF_Rest_ (%)	52(47, 57)	50(44, 53)	55(47, 59)	**<0.001**
TIDEDV	1.05 (0.96, 1.13)	1.05 (0.97, 1.14)	1.04 (0.95, 1.12)	0.400
TIDESV	1.08 (0.97, 1.22)	0.98 (0.91, 1.07)	1.14 (1.02, 1.26)	**<0.001**
ΔLVEDV (ml)	4(−4, 10)	5(−2, 12)	4(−4, 10)	0.463
ΔLVESV (ml)	3(−1, 8)	−1 (−6, 3)	5(1, 10)	**<0.001**
Angiographic findings, *n* (%)				0.650
1-vessel	116(0.45)	35(0.46)	81(0.44)	
2-vessel	83(0.32)	26(0.34)	57(0.31)	
3-vessel	61(0.23)	15(0.20)	46(0.25)	
Left main coronary disease	10(0.04)	3(0.04)	7(0.04)	0.957
Treatment strategy, *n* (%)				0.342
Conservative strategy	111(0.43)	29(0.38)	82(0.45)	
Invasive strategy	149(0.57)	47(0.62)	102(0.55)	
Baseline medication, *n* (%)				
Aspirin	251(0.97)	72(0.95)	179(0.97)	0.517
Statin	251(0.97)	72(0.95)	179(0.97)	0.517
Beta-blocker	193(0.74)	56(0.74)	137(0.75)	0.897
Calcium channel blocker	72(0.28)	20(0.26)	52(0.28)	0.750
ACE inhibitor or ARB	113(0.44)	30(0.40)	83(0.45)	0.404

LVEDV, left ventricular end-diastolic volume; LVESV, left ventricular end-systolic volume; LVEF, left ventricular ejection fraction; TID, transient ischemic dilatation, ΔLVEDV = LVEDV_Stress_ - LVEDV_Rest_, ΔLVESV = LVESV_Stress_ - LVESV_Rest_; ACE, angiotensin-converting enzyme; ARB, angiotensin receptor blocker; MACEs, major adverse cardiac events.

Significant *P*-values in bold.

An equivalent proportion of patients underwent exercise or pharmacological stress testing in the two groups (*P* = 0.229), and no significant differences were observed between the two groups in terms of TPD, scarring, ischemia, ischemia >10%, LVEDV_Stress_, LVESV_Stress_, LVEDV_Rest_, TIDEDV and ΔLVEDV. The ΔLVEF ≤ 0% group exhibited a higher LVEF_Rest_ than the ΔLVEF > 0% group (*P* < 0.001), whereas LVEF_Stress_ was higher in the ΔLVEF > 0% group (*P* = 0.001). Patients with ΔLVEF ≤ 0% had a smaller LVESV_Rest_ (*P* = 0.001) and a greater TID-ESV (*P* < 0.001) and ΔLVESV (*P* < 0.001) than patients with ΔLVEF > 0%. There was no significant difference between the two groups regarding angiographic findings, treatment strategy, and medications.

### 3.2 Clinical outcomes

During a mean follow-up period of 33.4 ± 7.6 months, we recorded 69 MACEs (26.5%), including 10 all-cause deaths, 2 myocardial infarctions, 28 coronary revascularizations, and 29 hospitalizations for unstable angina. The ΔLVEF ≤ 0% group had a significantly increased event rate for the primary endpoint of MACEs (*P* = 0.027). However, when individual MACEs were analysed separately, no significant differences were observed between the two groups (*P* > 0.05) (Table 2).

**Table 2:** Major adverse cardiac events by left ventricular ejection fraction reserve group.

	All	ΔLVEF > 0%	ΔLVEF ≤ 0%	*P*-value
	*n* = 260	*n* = 76	*n* = 184	
Total MACEs, *n* (%)	69 (0.27)	13 (0.17)	56 (0.30)	**0.027**
All-cause death	10 (0.04)	2 (0.03)	8 (0.04)	0.764
Myocardial infarction	2 (0.01)	0	2 (0.01)	0.895
Coronary revascularization	28 (0.11)	5 (0.07)	23 (0.13)	0.161
Hospitalization for unstable angina	29 (0.11)	6 (0.08)	23 (0.13)	0.283

MACEs, major adverse cardiac events.

Significant *P*-values in bold.

**Figure 2:**
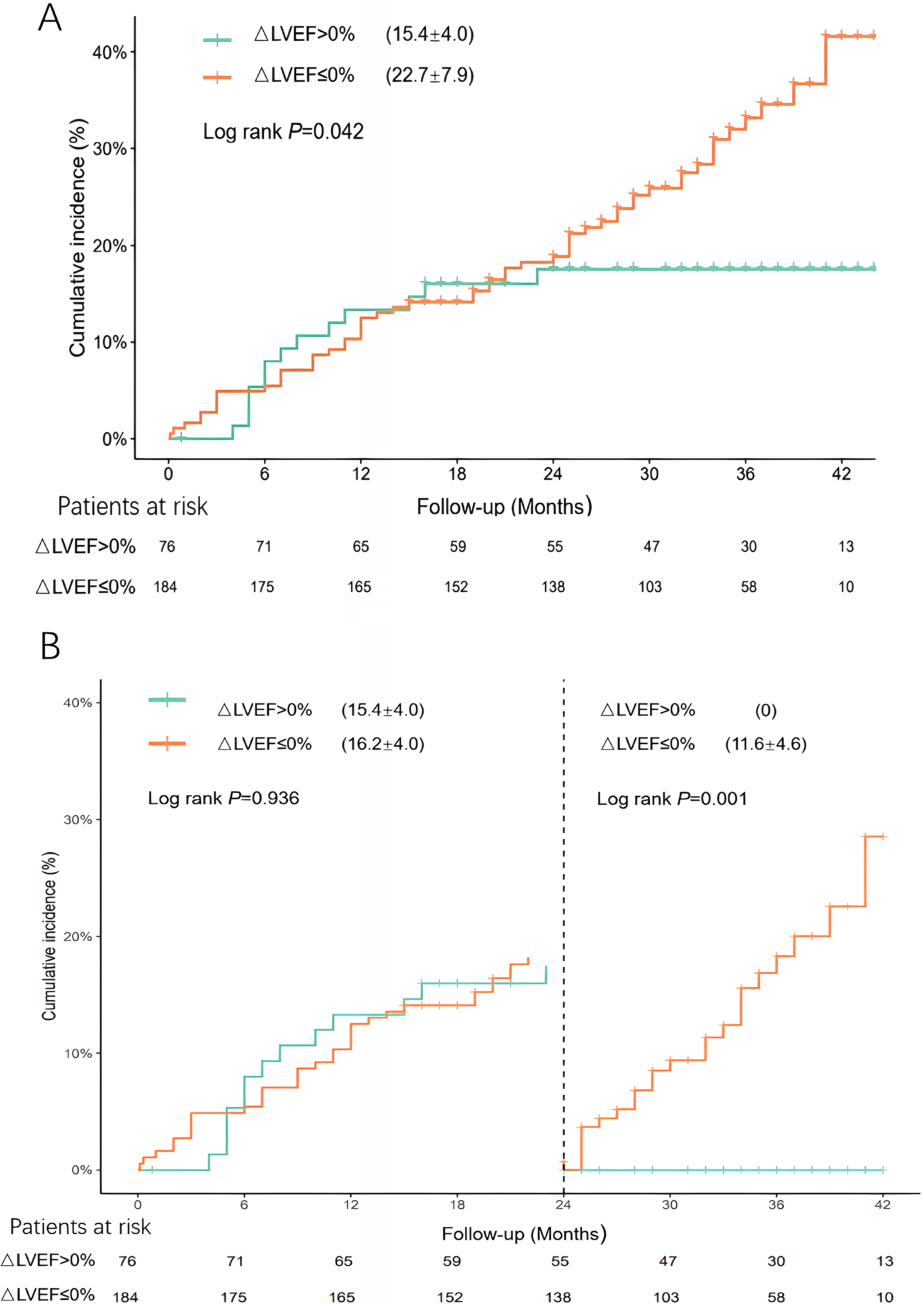
**(A)** Cumulative incidence of MACEs in patients with different LVEF reserves. **(B)** Landmark analyses were performed using a landmark point of 2 year and beyond 2 years.

As depicted in Figure 2A, the cumulative incidence of MACEs in patients with an ΔLVEF of ≤ 0% (22.7% ± 7.9%) was significantly higher than that in patients with an ΔLVEF >0% (15.4% ± 4.0%) (*P* = 0.042). Landmark analysis was performed at 2 years and beyond 2 years (Figure 2B). At 2 years, there was no significant difference in cumulative incidence of MACEs between two groups. Beyond 2 years, the cumulative incidence of MACEs in the ΔLVEF ≤ 0% group (11.6% ± 4.6%) was significantly higher than that in the ΔLVEF > 0% group (0%) (*P* = 0.001). In addition, considering the guideline (23) by The British Society of Echocardiography, a “normal” LVEF is defined as ≥55%, the sensitivity analysis was conducted using a 55% as a cutoff point. We compared the cumulative incidence of MACEs between the two groups in patients with LVEF_Stress_ < 55% (*n* = 181) and LVEF_Rest_ < 55% (*n* = 160). In patients with LVEF_Stress_ < 55%, the cumulative incidence of MACEs revealed differences, but these did not reach statistical significance (*P* = 0.188). In patients with LVEF_Rest_ < 55%, the differences achieved statistical significance (*P* = 0.045) (Supplementary Figure S1 and Supplementary Figure S2).

**Figure 3:**
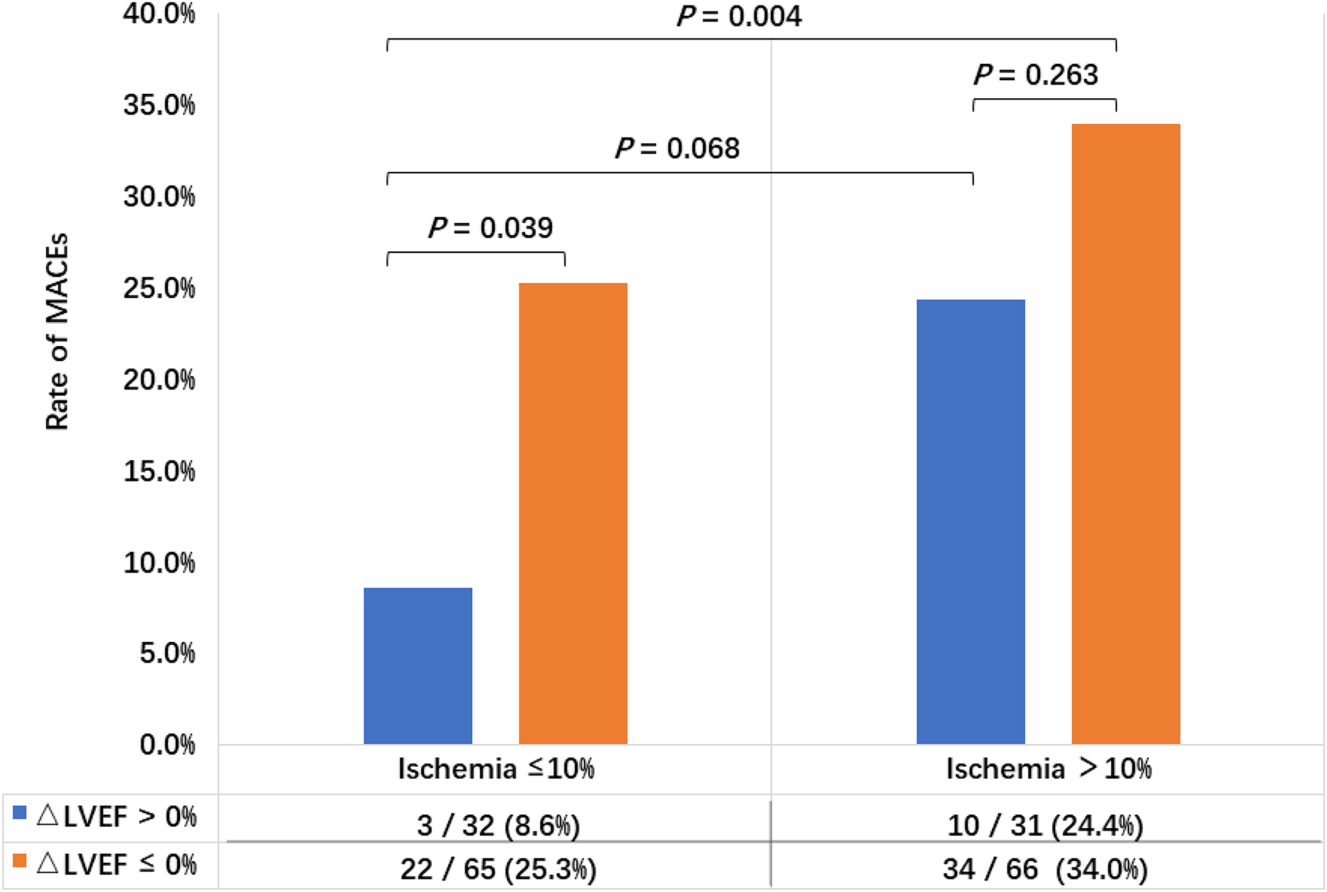
Comparison of the incidence of MACEs between different LVEF reserves in patients with no or mild myocardial ischemia (extent of ischemia ≤ 10%) and moderate to severe myocardial ischemia (extent of ischemia > 10%).

Figure 3 compares the rate of MACEs between different LVEF reserves in patients with no or mild myocardial ischemia (extent of ischemia ≤ 10%) and moderate to severe myocardial ischemia (extent of ischemia > 10%). In patients with no or mild myocardial ischemia, the incidence of MACEs in the ΔLVEF ≤ 0% group (25.3%) was significantly higher than that in the ΔLVEF >0% group (8.6%) (*P* = 0.039). However, no significant difference was detected between the LVEF reserve groups in patients with moderate to severe myocardial ischemia (*P* = 0.263).

### 3.3 MACE prediction by univariate and multivariate Cox regression analysis

Univariate Cox regression analysis revealed that TPD, an extent of ischemia > 10%, LVESV_Stress_, ΔLVEF ≤ 0%, and multivessel disease were all independent predictors for MACEs. However, LVEF_Stress_ and LVEF_Rest_ were identified as independent negative predictors. Multivariate Cox analysis showed that LVEF_Stress_ [adjusted HR: 0.972; 95% CI: 0.949, 0.995, *P* = 0.016] was an independent negative predictor while an ΔLVEF ≤ 0% [adjusted HR: 1.276; 95% CI: 1.006, 1.618, *P* = 0.045] was an independent positive predictor of MACEs (Table 3).

**Table 3:** Univariate and multivariate Cox regression analysis for MACEs.

	Univariate Cox regression analysis for MACEs					Multivariate Cox regression analysis for MACEs				
	Hazard ratio	95% CI			*P* value	Hazard ratio	95% CI			*P*-value
Age	1.006	0.982	–	0.982	0.646					
Male	1.326	0.712	–	0.712	0.374					
Body mass index	1.041	0.970	–	0.970	0.261					
Hypertension	0.952	0.577	–	0.577	0.846					
Diabetes	1.148	0.712	–	0.712	0.572					
Hyperlipidemia	1.316	0.808	–	0.808	0.269					
Current smoker	0.874	0.523	–	0.523	0.607					
Previous infarction	1.084	0.633	–	0.633	0.769					
Previous revascularization	1.336	0.817	–	0.817	0.248					
Total perfusion defect	1.016	1.002	–	1.030	**0.024**	1.001	0.982	-	1.020	0.929
Scar extent	1.009	0.993	–	1.025	0.258					
Ischemia extent	1.015	0.997	–	1.033	0.111					
Ischemia extent > 10%	1.789	1.093		2.929	**0.021**	1.608	0.098	–	2.645	0.061
LVEDV_Stress_	1.005	1.000	–	1.011	0.068					
LVESV_Stress_	1.007	1.000	–	1.013	**0.035**	0.999	0.986	–	1.011	0.854
LVEF_Stress_	0.964	0.943	–	0.985	**0.001**	0.972	0.949	–	0.995	**0.016**
LVEDV_Rest_	1.004	0.998	–	1.010	0.178					
LVESV_Rest_	1.005	0.998	–	1.012	0.191					
LVEF_Rest_	0.974	0.952	–	0.995	**0.018**					
TIDEDV	1.483	0.301	–	7.322	0.628					
TIDEDV ≥1.2	0.791	0.362	–	1.729	0.557					
TIDESV	1.566	0.539	–	4.554	0.410					
TIDESV ≥1.2	0.819	0.474	–	1.416	0.474					
ΔLVEDV	1.005	0.986	–	1.024	0.605					
ΔLVESV	1.017	0.992	–	1.042	0.186					
ΔLVEF	0.976	0.938	–	1.015	0.228					
ΔLVEF ≤ 0%	1.321	1.042	–	**1.676**	**0.022**	1.276	1.006	–	1.618	**0.045**
Multi-vessel disease	1.872	1.134	–	**3.090**	**0.014**	1.573	0.945	–	2.616	0.081
Aspirin	0.427	0.155	–	1.172	0.098					
Statin	1.087	0.266	–	4.438	0.907					
Beta blocker	0.836	0.497	–	1.407	0.501					
Calcium channel blocker	1.157	0.693	–	1.932	0.578					
ACE inhibitor or ARB	1.104	0.688	–	1.773	0.681					
Invasive strategy	0.886	0.552	–	1.422	0.617					

Significant *P*-values in bold.

### 3.4 Incremental prognostic value of LVEF reserve

**Figure 4:**
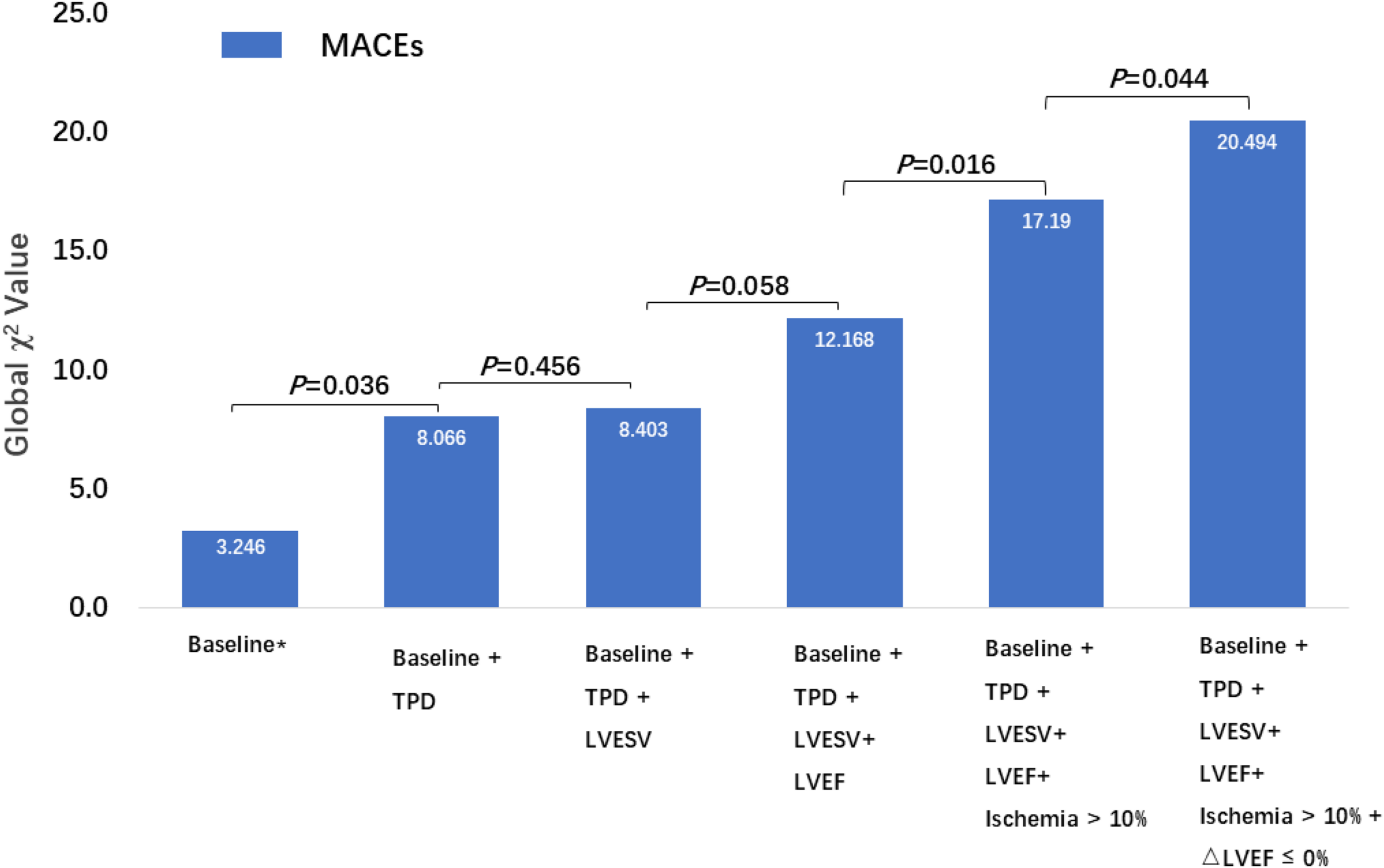
Incremental prognostic value of MPI variables, including TPD, LVESV_Stress_, LVEF_Stress_, an extent of ischemia >10%, and LVEF reserve, for MACEs in patients with CAD and with a LVEF_Stress_ < 60%. * Baseline including age, sex and BMI.

**Figure 5:**
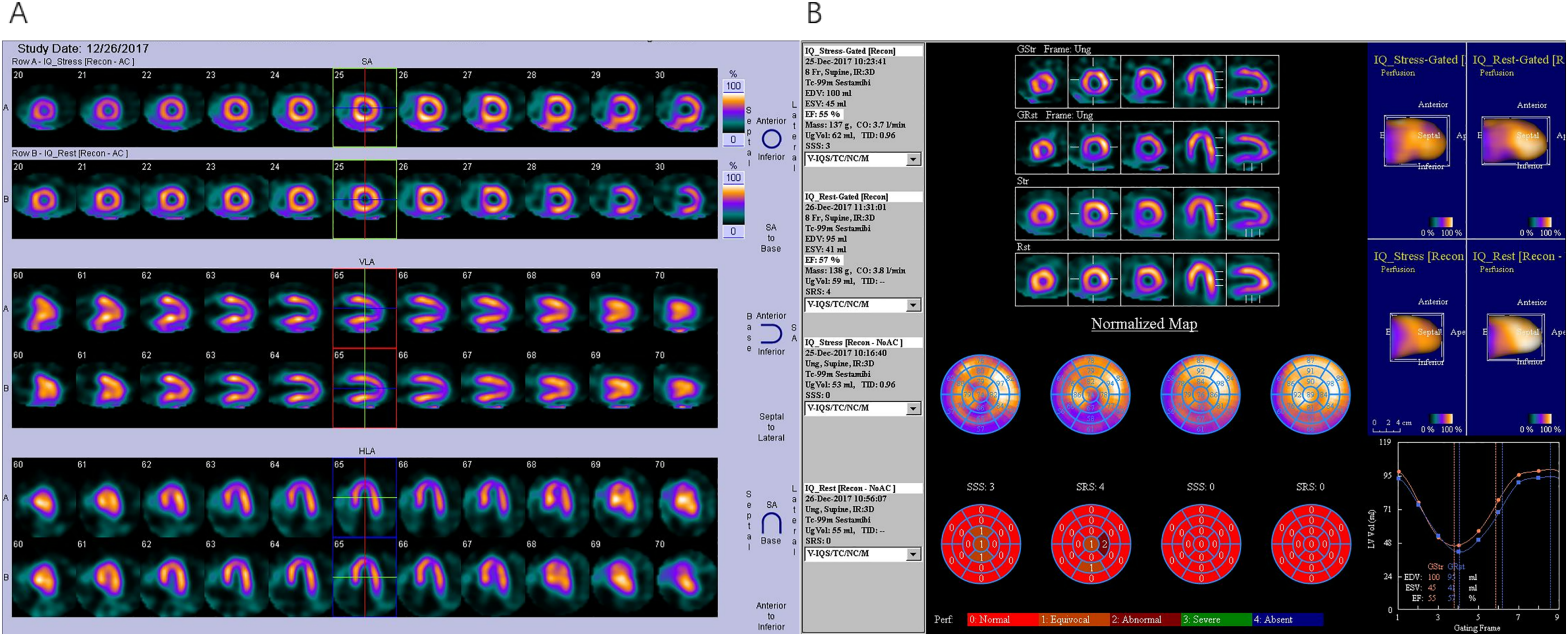
SPECT G-MPI in a 61-year-old male with CAD and with a history of PCI. **(A)** Perfusion imaging showing mild stress-induced ischemia in the apical inferior. **(B)** Analysis of cardiac function parameters revealed a LVEF_Stress_ of 55%, a LVEF_Rest_ of 57%, and a ΔLVEF = LVEF_Stress_ – LVEF_Rest_ = −2%. Subsequent coronary angiography revealed the absence of significant stenosis in the LM and LAD, patency of stent in LCX, and 100% occlusion of RCA. After a failed attempt of PCI in RCA, the patient was given medical therapy, and acute myocardial infarction was detected after 2.5 years of follow-up. PCI, percutaneous coronary intervention; LM, left main stem; LAD, left anterior descending artery; LCX, left circumflex artery; RCA, right coronary artery.

Figure 4 illustrates the global *χ*^2^ value for the prediction of MACEs. The global *χ*^2^ for Model 2 (Baseline + TPD) increased significantly from Baseline (Age, Sex and BMI, *P* = 0.036). The global *χ*^2^ for Model 3 (Model 2 + LVESV_Stress_) did not significantly improve the prediction of MACEs (*P* = 0.456). The trend of an increase in global *χ*^2^ for Model 4 (Model 3 + LVEF_Stress_) compare to Model 3 was observed but did not reach statistical significance (*P* = 0.058). The global *χ*^2^ for Model 5 (Model 4 + the extent of ischemia > 10%) was significantly higher than that for Model 4 (*P* = 0.016). Finally, the global *χ*^2^ for Model 6 (Model 5 + ΔLVEF ≤ 0%) was significantly higher than that for Model 5 (*P* = 0.044). A typical case is presented in Figure 5.

## 4 Discussion

This study aimed to evaluate the prognostic value of ΔLVEF, as determined by SPECT G-MPI in patients with CAD, to predict MACEs. Our results indicated that in patients with a LVEF_Stress_ < 60%, an *Δ*LVEF ≤ 0% was identified as independent predictors of MACEs by multivariate Cox regression analysis. Furthermore, in patients with no or mild myocardial ischemia, the incidence of MACEs in the ΔLVEF ≤ 0% group was significantly higher than in the ΔLVEF > 0% group. Moreover, adding ΔLVEF to the traditional perfusion and functional variables of MPI significantly improved the discriminatory power to predict MACEs. Our results were generally consistent when left ventricular systolic dysfunction was defined as LVEF_Rest_ < 55%.

LVEF has been a key variable for the diagnosis and management of heart failure. In our study, we specifically focussed on LVEF_Stress_ < 60% because the latest recommendations by the British Society of Echocardiography (14) and the American Society of Nuclear Cardiology (24) state that the cut-off value for a “normal” LVEF is 55%. However, differences have been identified in terms of sex, age, and ethnicity. For example, there is a clear difference in LVEF between Europeans and Asians. The predicted values for Europeans are known to be significantly lower than those for East Asians. Specifically, for both sexes (at the age of 50 years), the lower reference value of LVEF for Europeans was 6% lower than that for East Asians. Furthermore, tenfold more Europeans than East Asians were found to have an LVEF < 50% (25). Unfortunately, while the LVEF criteria are applicable and appropriate for European populations, there is a significant scarcity of available data relating to LV function parameters acquired by gated MPI in the Chinese population. In addition, we cannot ignore the wide limits of agreement between echocardiography and SPECT G-MPI when determining LVEF (26). In routine clinical practice, we recommend monitoring borderline LVEF to avoid delay or missing high-risk patients. Our recent study (15) provided insights into the normal reference values of LVEF_Stress_ when measured by D-SPECT G-MPI in both women and men, which were 70 ± 8% and 68 ± 7%, respectively. Therefore, our centre gives considerable attention to patients with LVEF_Stress_ < 60%. Meanwhile, the results were generally consistent when left ventricular systolic dysfunction was defined as LVEF_Rest_ < 55%, but not when defined as LVEF_Stress_ < 55%. Indeed, in some centres, the stress-only strategy, or stress-first strategy, has been implemented to reduce costs and enhance the efficacy of testing (27, 28). Therefore, an appropriate expansion of the criteria for LVEF_Stress_ reduction aligns with clinical practice.

Previous evidence showed that a reduced ΔLVEF, as determined by 82Rb PET MPI, serves as a marker for ischemic contractile dysfunction (7) and is associated with an increased risk of cardiac events (5) and all-cause mortality (18). However, the existing literature describes inconsistent findings concerning the predictive significance of *Δ*LVEF when determined by SPECT MPI (9, 10, 12). In a previous study, Smith et al. (12) demonstrated that an abnormal LVEF reserve was not associated with an increased risk of the primary outcome. One possible explanation for this difference is that most patients in the study reported by Smith et al. (12) underwent a single-day protocol. In contrast, a two-day protocol was used in the present study as per our routine clinical practice. In the single-day protocol, the rest examination was performed approximately three hours after the stress examination, possibly leading to an underestimation of the alteration in LVEF, particularly in patients with severe ischaemia who may have experienced prolonged stunning. In addition, our landmark analysis revealed the effect of *Δ*LVEF on long-term prognosis. Specifically, in our cohort of patients with coronary stenosis and left ventricular systolic dysfunction, 23 MACEs were observed beyond 2 years, a large proportion (78.3%) of whom underwent incomplete revascularization (*n* = 2) and conservative strategies (*n* = 16). BARI-2D (29) found in high-risk patients, including those with reduced LVEF and extensive coronary disease, the five-year risk of death/MI/stroke were significantly lower among those undergoing revascularizations when compared with the group of medical therapy alone. In particular, the survival curve showed a significant increase in the difference in event rates after 2 years. Similarly, STICH trial (30) reported a significant benefit began to accrue after 2 years when comparing CABG and medical therapy in patients with heart failure. Our results strongly correlated with the above reports. We speculate that the absence of LVEF reserve may indicate a declining cardiac reserve, and that coronary artery stenosis and progressive myocardial ischemia may contribute to this poor prognosis in the later stages.

In a previous study, Gomez et al. (9) defined an abnormal *Δ*LVEF as a reduction of <5% in LVEF in post-stress images. This criterion was derived from a previous study (31) that proposed a 5% threshold for *Δ*LVEF when distinguishing between normal and abnormal responses. The study demonstrated that a *Δ*LVEF of 5% provided the highest diagnostic accuracy (sensitivity 52%, specificity 83%) for detecting multivessel CAD. Nevertheless, the most extensive cohort study to date (10), featuring 10,275 patients who underwent SPECT-MPI, revealed that an increase of 1% in LVEF reserve was significantly and independently associated with a lower incidence of MACEs, including cardiac death and myocardial infarction [HR: 0.98; 95% CI: 0.97, 0.99, *P* = 0.003]. Thus, additional clarification is needed to enable a more significant prognostic capability for patient outcomes. Within our present cohort, only 9.2% (*n* = 24) of patients exhibited an ΔLVEF of ≥5%, thus indicating that an ΔLVEF of ≤0%, rather than an ΔLVEF of ≤5%, represents a crucial and autonomous prognostic marker, thereby aligning with recent research (11, 18, 19). However, the prognostic value of ΔLVEF, as determined by SPECT MPI, has not been reported in a high-risk cohort with a reduced LVEF. This study is the first to report that an ΔLVEF ≤ 0% was an independent predictor of MACEs in patients with a LVEF_Stress_ < 60%. This finding provides a valuable point of reference for guiding future clinical practice.

Previous research established the importance of myocardial ischemia for determining therapeutic strategies. Patients with no to mild ischemia were categorised as low risk, for whom a conservative treatment approach was considered to be appropriate. In contrast, patients with moderate to severe ischemia were recommended for revascularization to improve their prognosis (32, 33). To the best of our knowledge, there is a significant scarcity of data relating to the prognosis of *Δ*LVEF in patients with varying degrees of myocardial ischemia. Smith et al. (12) previously performed subgroup analysis for patients with large areas of ischemia (≥10%/LV) and determined no significant difference in the incidence of primary outcomes compared to those with and without LVEF reserve. These findings are consistent with those arising from our present analysis. Unfortunately, the study lacked data on patients with no to mild ischemia. Our results suggest, for the first time, that the combination of ΔLVEF with the extent of myocardial ischemia could enhance risk stratification in patients with CAD. Notably, patients with no to mild myocardial ischemia were considered to have a favourable prognosis, whereas those with an ΔLVEF ≤ 0% exhibited a relatively high risk of MACEs.

A large area of myocardial ischemia has been confirmed to be associated with poor outcomes in CAD patients. Its prognostic effect is very strong and significant. In our cohort, a total of 44 MACEs were observed in patients with moderate to severe myocardial ischemia, with 75% (*n* = 33) occurring within 2 years. Our results reveal that the influence of the ischemia on prognosis was significantly greater than that of ΔLVEF in a short term. The influence of ΔLVEF on outcomes has gradually become more apparent with the progression of the disease. In fact, the mechanism of the prognostic significance of ΔLVEF is not very clear at present. We observed that the ΔLVEF ≤ 0% group exhibited a higher LVEF_Rest_ than the ΔLVEF > 0% group, whereas LVEF_Stress_ was higher in the ΔLVEF > 0% group. Furthermore, the 79.5% of impaired LVEF_Stress_ (≤50%) (23) was included in the ΔLVEF ≤ 0% group (vs. ΔLVEF > 0% group, *P* = 0.005). In contrast, a slightly higher proportion of individuals with supra-normal left ventricular ejection fraction (snLVEF) (LVEF_Rest_ ≥ 65%) were found in the ΔLVEF ≤ 0% group (*n* = 9, *P* = 0.050), compared to ΔLVEF > 0% group (*n* = 0). The snLVEF is considered to be associated with a poor prognosis (34), but the mechanism is unclear. We speculate that the combination of potential functional abnormalities in the resting state and impaired cardiac reserve, which presents a poor response to stress, may lead to a reduced ΔLVEF and posing a risk of long-term poor prognosis. Further research into this potential relationship is needed.

To our knowledge, only one previous study investigated the incremental value of ΔLVEF for predicting MACEs beyond the conventional variables of MPI. Otaki et al. (11) recruited 151 patients undergoing same-day rest/stress SPECT G-MPI. Early stress imaging was initiated 2 min after the injection of regadenoson, followed by late-stress acquisition. This study demonstrated that adding ΔLVEF during early stress enhanced the combined model of age, a prior history of PCI, and TPD (*P* < 0.001). The annualised MACEs rates during the late-stress period exhibited variances between patients with an ΔLVEF < 0% (6.7%) and an ΔLVEF ≥ 0% (4.9%), although these differences were not statistically significant. However, the sample size of this previous study was limited and focused explicitly on preserved LVEF_Stress_, unlike our current study. Furthermore, Otaki et al. did not analyse the traditional parameters of MPI, except for TPD. It is widely acknowledged that larger perfusion defects, reduced ejection fraction, and larger ventricular volume predict adverse cardiac events (10, 35). Our current findings concur with these earlier findings. Based on our current findings, we emphasize that in patients with left ventricular dysfunction, both stress and resting MPI parameters, including TPD, ischemia, and LVEF, particularly ΔLVEF, may provide valuable assistance for the further risk stratification of patients with CAD.

The European Society of Cardiology guidelines (13) published recently for managing chronic coronary syndromes (CCS), guide clinicians in choosing imaging techniques (36). Both functional and anatomical aspects must be considered in patients with suspected CCS, and the importance of non-invasive imaging for selecting patients to be referred for invasive angiography has been emphasized. In particular, functional assessment may be crucial for identifying the mechanisms behind myocardial ischemia and, eventually, angina, thus guiding symptomatic treatment (37). Speckle tracking echocardiography (STE) is a reliable and widely used imaging technique of recognized clinical value in several settings. This method uses the motion of ultrasound backscatter speckles within echocardiographic images to derive myocardial velocities and deformation parameters (38). Notably, global longitudinal strain (GLS) is considered an earlier marker of myocardial damage and predicts mortality in patients with CCS independently of LVEF (39). The myocardial deformation imaging might reveal subtle abnormalities that can be attributed to clinically relevant ischemic or ischemic memory (40). This ischemic memory may be considered relevant to myocardial stunning and the reduction of post-stress LVEF (41). Integrating multiple imaging modalities and attempting to reveal the pathophysiological mechanisms is an important direction for future research.

Some studies support the notion that the presence of TID can specifically indicate extensive or severe coronary artery disease (42). However, this study found that TID was not an independent predictor for MACEs, thus aligning with a large cohort study previously conducted by Kattoor et al. (10) who found that the prognostic value of ΔLVEF was higher than that of TID. The pathophysiology of TID remains controversial (43, 44), although the predominant hypothesis is that TID originates from either diffuse subendocardial hypoperfusion leading to an apparent increase in LV endocardial cavity size and/or stress-induced LV dysfunction (3). Although investigating a specific group of patients with LV dysfunction may influence the prognostic value of TID, it is noteworthy that we identified clear differences between the ΔLVEF groups for ΔLVESV and TIDESV but not for ΔLVEDV or TIDEDV, thus indicating an association between a reduction in post-stress LVEF and left ventricular systolic dysfunction.

## 5 Limitations

Our research is subject to several limitations that need to be considered. First, owing to its retrospective nature and the fact that this was a single-centre study with a relatively small sample size, there is potential for selection bias. To reduce the waiting list time for MPI, we did not perform rest studies in patients with normal stress-gated MPI in our laboratory. Therefore, even if more than 3,000 MPI studies were conducted per year, the number of populations in the current study was limited. Another limitation was the acquisition of gated MPI, which was performed after 60–90 min according to different stress or rest states. This implies that the acquisition of LVEF_Stress_ by SPECT was not derived during peak stress. However, we confirmed the predictive value of ΔLVEF in patients with a LVEF_Stress_ < 60%, particularly in those with no or mild myocardial ischemia. Our findings emphasise that combining perfusion and cardiac function parameters may enhance risk stratification.

## 6 Conclusions

In this pilot study, we found that when determined by SPECT G-MPI, ΔLVEF was independently associated with MACEs in CAD patients with LVEF_Stress_ < 60%, enhancing risk stratification for MACEs. Patients with no to mild myocardial ischemia were considered to have a favourable prognosis, whereas those with an ΔLVEF ≤ 0% exhibited a relatively high risk of MACEs. This is a pilot study with a small sample size, and further investigation and validation are needed.

## Data Availability

The original contributions presented in the study are included in the article/Supplementary Material, further inquiries can be directed to the corresponding author.

## Supplementary material

Supplementary Figure S1 Cumulative incidence of MACEs in patients with different LVEF reserves in patients with LVEF_Stress_ < 55%.

Supplementary Figure S2 Cumulative incidence of MACEs in patients with different LVEF reserves in patients with LVEF_Rest_ < 55%.
