# Supplementary figures and images for: Prognostic value of the left ventricular ejection fraction reserve acquired by gated myocardial perfusion SPECT in patients with CAD and reduced stress LVEF
Source: Front Cardiovasc Med. 2024 Oct 10;11:1480501. doi: 10.3389/fcvm.2024.1480501 (PMC11499129; doi:10.3389/fcvm.2024.1480501)

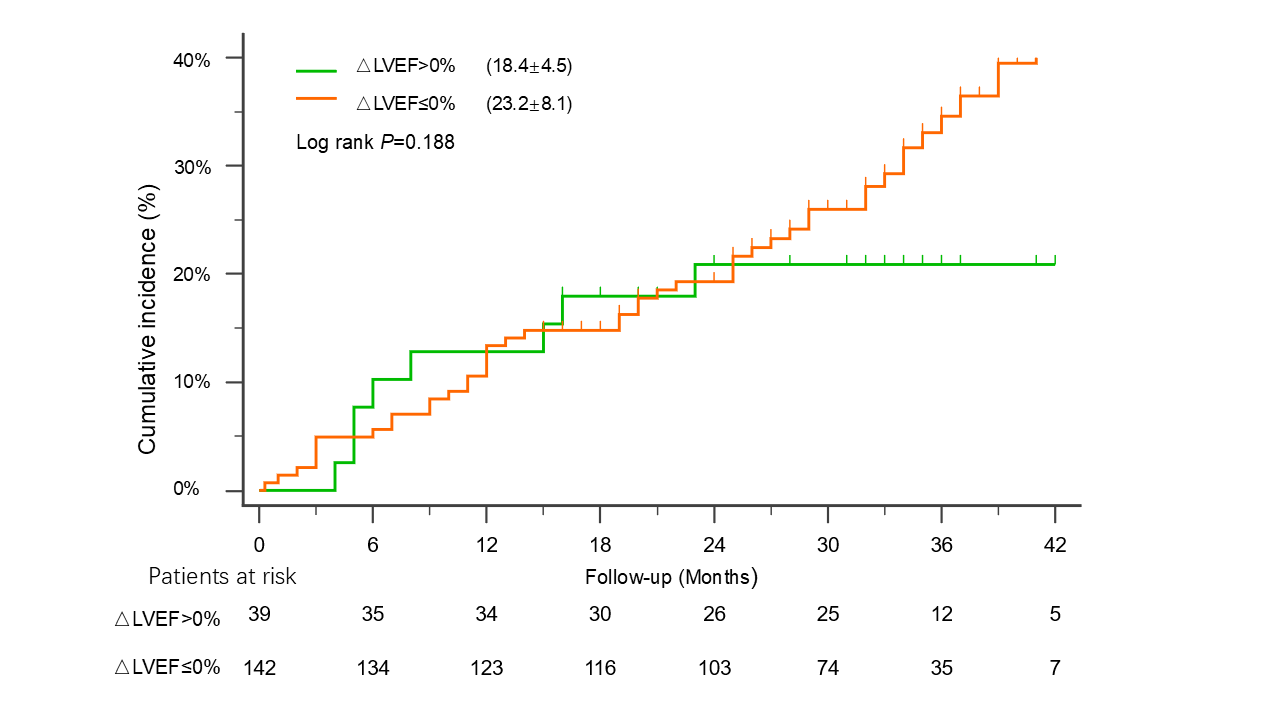

Supplement: Supplementary Figure S1 — Cumulative incidence of MACEs in patients with different LVEF reserves in patients with LVEFStress < 55%. [file Image1.tif]

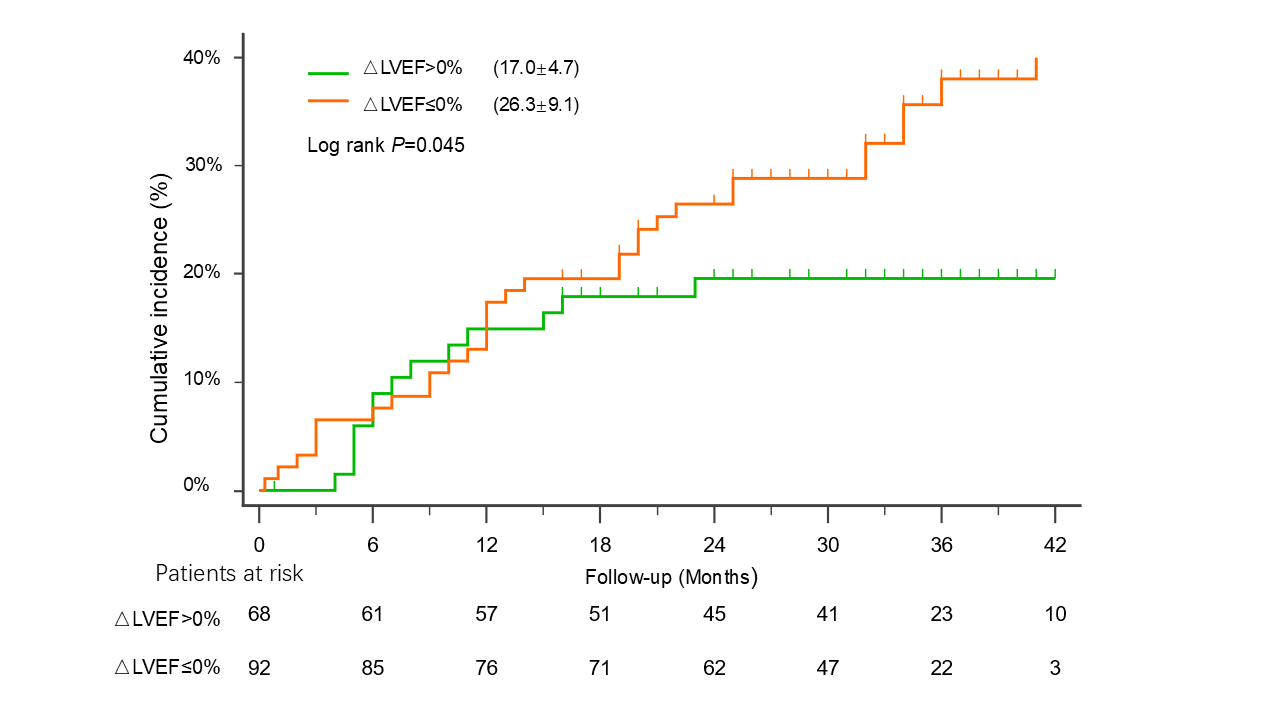

Supplement: Supplementary Figure S2 — Cumulative incidence of MACEs in patients with different LVEF reserves in patients with LVEFRest < 55%. [file Image2.tif]
